# The mental health and wellbeing of healthcare workers during COVID-19 in South Africa

**DOI:** 10.4102/hsag.v28i0.2159

**Published:** 2023-03-31

**Authors:** Jennifer Watermeyer, Sonto Madonsela, Johanna Beukes

**Affiliations:** 1 Health Communication Research Unit, School of Human and Community Development, Faculty of Humanities, University of the Witwatersrand, Johannesburg, South Africa

**Keywords:** COVID-19, experience, healthcare worker, mental health, qualitative, South Africa

## Abstract

**Background:**

Little is known about the experiences and impact of coronavirus disease 2019 (COVID-19) on the mental health and wellbeing of healthcare workers (HCWs), particularly in Global South contexts.

**Aim:**

The authors aimed to explore the experiences of HCWs at different points during the COVID-19 pandemic in South Africa.

**Setting:**

This study’s sample included 621 HCWs from various professions and health sectors who completed the survey during the pandemic peaks of waves I, II and III in South Africa.

**Methods:**

The authors used a qualitative survey design exploring participants’ general work, life, mental health and wellbeing experiences, and their support mechanisms or strategies. Data were analysed using thematic analysis.

**Results:**

The authors identified three overarching themes in the data, namely stress, adjustment to work during COVID-19, and support experiences and needs. These themes were common across all three survey waves, with some minor differences noted across the waves.

**Conclusion:**

An overarching thread of uncertainty seems central to HCWs’ experiences of working during COVID-19, related to pressures in the South African healthcare system that have been aggravated by the pandemic.

**Contribution:**

These findings have the potential to inform the development of contextually relevant approaches to support the mental health and wellbeing needs of HCWs during and after a pandemic. In particular, workplaces need to actively offer psychological support to all HCWs, not just to workers traditionally defined as frontline.

## Introduction

Healthcare work is often associated with poor wellbeing and burnout (Hall et al. 2016; Johnson et al. 2018). The coronavirus disease 2019 (COVID-19) pandemic has added to healthcare workers’ (HCWs’) stress in significant ways, as borne out by the numerous reviews on this topic (De Kock et al. 2021; Greenberg 2020; Saragih et al. 2021; Spoorthy, Pratapa & Mahant 2020). Chutiyami et al.’s (2021) meta-review of systematic reviews indicates anxiety, depression and stress/post-traumatic stress disorder as most prevalent among HCWs from all sectors across the globe during the pandemic. Importantly, although there may be similarities in HCWs’ experiences during the pandemic, research has also shown heterogeneity among responses (Grailey, Lound & Brett 2021).

Although much research has been conducted on HCWs during COVID-19 internationally, little attention has been given to HCWs’ experiences in contexts in the Global South such as South Africa where the pandemic has had particular consequences. A lack of resources and high poverty rates resulted in difficulties in many communities with adherence to social distancing regulations, increased health risks from patients with tuberculosis (TB) and HIV, and significant disparities between public and private healthcare systems. Corruption has been rife and extreme lockdowns resulted in a significant socio-economic impact on communities. The pandemic placed additional pressure on an already under-resourced health service, across both the public and private sectors but particularly affecting the public sector. During the peaks of waves I–III of the pandemic, hospitals across the country could not cope with the influx of COVID-19-positive patients (Burger & Mchenga 2021; Van Den Heever et al. 2021).

A handful of studies have focused on the experiences of South African HCWs during the pandemic, but to our knowledge, none have explored qualitative aspects of HCW experiences across a broad spectrum of occupations nor have these studies focused on experiences across the pandemic. Naidoo et al. (2020) conducted a survey with a range of allied and medical South African HCWs at the start of the pandemic in early 2020, concluding that psychological distress was highest among nurses. Dawood, Tomita and Ramlall (2022) conducted a similar survey in the second half of 2020 with a sample predominantly including doctors. They reported high levels of depression, anxiety, stress and traumatic stress together with poor perceptions of support from employers. Curran et al.’s (2021) survey conducted primarily with nurses in one district during the second half of 2020 concluded that psychological support is needed for HCWs and especially for managers in healthcare institutions. Wooyoung, Maaroganye and Subramaney (2021) conducted a mixed-method study among psychiatric healthcare providers in one province in 2020 and the early part of 2021, reporting elevated psychiatric morbidity among participants. Most of these South African studies have in common a quantitative rather than a qualitative focus.

To provide appropriate, contextually relevant support to HCWs, there is a need to understand their experiences of working during this pandemic in relation to mental health and wellbeing. The data reported in this article follows a mixed-method embedded design as part of a larger non-experimental, correlational survey study that sought to understand the psychological states, resilience and coping mechanisms or strategies of South African HCWs at various points during the pandemic. The larger study drew on qualitative and quantitative methods including validated psychometric tests and open-ended questions. This article reports on the qualitative findings from that study which aimed to explore the experiences of South African HCWs working during the pandemic, focusing on exploring participants’ general work, life, mental health and wellbeing experiences, and their support mechanisms or strategies.

## Materials and methods

### Design

A qualitative descriptive design (Sandelowski 2000) was chosen to obtain a broad range of responses and to obtain a comprehensive picture of experiences from participants. The research team included two registered health professionals (Author 1 and Author 3) and a research psychologist (Author 2).

The authors collected data from HCWs using an anonymous online survey during the peaks of waves I, II and III of the COVID-19 pandemic in South Africa (around the periods June/July 2020, January 2021 and June/July 2021). The survey was open for approximately 2–3 weeks during each data collection period.

### Sampling strategy

The authors used a non-probability convenience sampling strategy. While the literature primarily describes HCWs as health professionals (doctors, nurses, psychologists, etc.), in this study, the authors defined the term more broadly to include anyone involved in healthcare work and/or working at a healthcare centre. The survey link was distributed to various allied health and medical professional bodies and also via social media networks.

### Instrument

A detailed survey was administered consisting of: a demographic section; scales to measure levels of depression, anxiety, burnout, resilience and coping strategies (not reported on in this paper) and six open-ended qualitative questions. The questions asked about experiences of work since the outbreak of COVID-19 in South Africa, physical health, support mechanisms or strategies, challenges at work and any comments for the Minister of Health during the current situation. Some of the questions were modified slightly across the three waves (Table 1 details the questions asked). The authors adopted guidelines for qualitative surveys from Braun et al. (2021).

**TABLE 1:** Questions used across the phases of data collection.

Question number	Wave 1	Wave 2	Wave 3
Q1	Briefly describe your experience of work since the COVID outbreak started in South Africa.	-	-
Q2	How has your health been over the last 2 weeks?	-	-
Q3	What support mechanisms do you currently have at work?	Please describe your support mechanisms at work – what has improved and what needs to improve?	-
Q4	What support mechanisms do you currently have at home?	-	Please describe your support mechanisms at home – what has made things easier for you and what still needs to be done to better support you at home?
Q5	What challenges are you currently experiencing in your work?	If you had to reflect on the last few months since the start of the pandemic, what lessons have been learnt – going forward what would need to be done differently?	-
Q6	If you could tell the Minister of Health one thing right now, what would it be?	-	-

COVID, coronavirus disease.

### Data analysis

Open-ended questions were analysed using thematic analysis principles and a coding reliability approach described by Braun et al. (2019). Coding was carried out on a question-by-question basis (i.e. comparing responses across participants) by Author 2 and Author 3 using Word and Excel platforms. Thereafter, results were consolidated into main themes that the entire research team identified and discussed using a consensus approach. To determine the saturation of themes, the authors conducted a broad tally across the responses.

### Trustworthiness

Trustworthiness was achieved in this study in several ways, guided by Shenton’s (2004) suggestions. Credibility was achieved via a peer debrief process during and after the analytic process. As this study forms part of a larger project there were opportunities for triangulation of our findings with results from the other study components. Transferability was achieved by describing the research context and assumptions central to the research. Dependability was achieved by keeping a running account of the research process of the project. Confirmability was achieved via careful checking of data sources and analytic findings and an audit trail of decision-making among the research team. The Standards for Reporting Qualitative Research (SRQR) checklist was followed.

### Ethical considerations

An application for full ethical approval was made to the University of the Witwatersrand’s Medical IRB, and ethical consent was received on 16/04/2020. The ethical approval number is M200461. All procedures performed in studies involving human participants were in accordance with the ethical standards of the institutional and/or national research committee and with the 1964 Helsinki Declaration and its later amendments or comparable ethical standards.

Participants were provided with an information sheet detailing the study. Completion of the survey was taken to mean consent. Participants were assured of confidentiality and anonymity.

## Results

### Response rates and demographic characteristics

The number of participants who chose to complete the qualitative parts of the broader survey included 405 (wave I), 124 (wave II) and 92 (wave III). The broader survey received a total of 766 responses across the three waves but only 621 participants (81%) chose to answer both the qualitative sections of the survey. The authors observed a sharp decrease in participation across the waves, particularly between waves I and II despite utilising the same recruitment strategies across all three waves.

Table 2 provides relevant demographic details of the participant sample. Participants were aged between 22 and 74 years (average M = 38.91, average SD = 11.133) and had been practicing for anything between 11 months to 54 years (average M = 14.54, average SD = 10.706). The majority of participants in the sample identified as female (*n* = 549, 88.4%) and spoke English as a home language (*n* = 391, 63%). In the datasets for waves I and III, the majority of participants were allied HCWs (72.1% and 80.4%, respectively), while the wave II dataset included a more even spread of medical (33.9%) and allied HCWs (27.4%). During wave I, just less than half of the participants reported having contact with COVID-19-positive patients, and this figure increased to just over half of the sample for waves II and III; the authors classified these participants as frontline HCWs regardless of whether they were directly treating COVID-19-positive patients or not.

**TABLE 2:** Sample demographic characteristics.

Variable	Wave I	Wave II	Wave III
**Age**			
Mean	38.19	40.70	37.86
Standard deviation	11.275	11.058	11.066
**Years of practice**			
Mean	14.364	14.641	14.603
Standard deviation	10.8935	9.8349	11.3901
**Gender† (%)**			
Female	91.6	80.6	84.8
Male	7.4	18.5	10.9
Other	0.2	0	1.1
**Home language‡ (%)**			
Afrikaans	27.6	20.2	34.8
English	65.6	62.1	59.8
IsiXhosa	1.2	4.0	1.1
IsiZulu	1.7	1.6	1.1
Sepedi	2.0	0.8	1.1
Sesotho	0.2	2.4	0
Setswana	0.5	5.6	1.1
SiSwati	0.2	0.8	0
Tshivenda	0.2	0	0
Xitsonga	0.2	0.8	0
**Occupation (%)**			
Allied$	72.1	27.4	80.4
Medical¶	13.1	33.9	9.8
Psychology/psychiatry	4.9	15.3	0
Dentist	4.0	0.8	0
Nurse	3.0	8.9	5.4
Pharmacist	2.0	0.8	0
Other††	1.0	12.9	4.4
**Frontline‡‡ (%)**			
No	53.1	30.6	44.6
Yes	46.9	69.4	55.4

Note: *N* = 405 (wave I), 124 (wave II) and 92 (wave III), except where indicated otherwise.

† , *N* = 403 (wave I), 123 (wave II) and 89 (wave III);

‡ , *N* = 396 (wave I), 122 (wave II) and 91 (wave III); $, ‘Allied’ includes audiologist, biokineticist, dietician, emergency care practitioner, occupational therapist, paramedic, physiotherapist, radiographer, social worker and speech-language therapist;

¶ , ‘Medical’ includes anaesthetist, cardiothoracic surgeon, clinical associate, community service doctor, doctor, embryologist, family physician, general practitioner, intern, medical officer, medical specialist, neonatologist, obstetrician and gynaecologist, ophthalmologist, paediatrician, pathologist, physician, radiologist, registrar and specialist physician;

†† , ‘Other’ includes clinical manager, environmental health practitioner, homeopath, health and safety manager, hospital case manager, hospital CEO, hospital manager, station commander and study coordinator;

‡‡ , ‘Frontline’ means the participant had contact with a COVID-positive patient regardless of whether they were directly treating the person or not.

### Themes identified in the data

The authors identified three overarching themes across the qualitative responses, namely: (1) stress, (2) adjustment and (3) support experiences and needs. These themes were common across all three survey waves, with some minor differences noted, although in general the major themes remained salient across the data sets. Importantly, these themes cannot be considered standalone themes, as they overlap and influence each other. Table 3 provides details of these major themes, subthemes and codes identified in the data.

**TABLE 3:** Qualitative themes, sub-themes and codes.

Major theme	Sub-themes	Codes
Stress related to working during COVID-19	Infection anxiety	Working with COVID-positive patients and colleagues
		Safety procedure adherence (patients and colleagues)
		Fear of exposing family and patients
		Lack of PPE and resource constraints
	Financial strain	Loss of income
	Poor mental health	Uncertainty, anxiety Decrease in optimal performance
	Poor physical health	Headaches, fatigue and poor sleep Existing medical conditions Unhealthy eating Testing COVID-19 positive
Adjustment to working during COVID-19	New protocols	Working with PPE
		Learning new protocols/regulations
	Uncertainty	Learning to live and work with uncertainty; need to be flexible and adapt
	Online therapy	Not connecting with patients
		Teletherapy is harder than in-person treatment
		Adjusting to teletherapy
	Workload	Increased workload
		Reduced workload
		More administrative work
Support experiences and needs	Support at work	Colleagues
		Debrief sessions
		Employee wellness programme
		Supportive management
		Peer supervision
		Professional organisations
		PPE
	Support at home	Family (including significant others)
		Friends
		Domestic help
		Pets
		Hobbies
	Other support networks	Church/religion
		Social networking
		Professional support (therapy)
	No support	No support from management
		Sole practice owner
		Physical support but no emotional support
		Lack of PPE
		Frontline workers supported but not other HCWs
		No support at home

PPE, Physical support; COVID-19, coronavirus disease 2019; HCWs, healthcare workers.

### Stress

Stress related to the changes brought about by the pandemic was a central theme in HCW’s experiences of working during COVID-19 – particularly stress about infecting others and loss of income, leading to physical and mental health challenges.

Especially during wave I, participants reported stress arising from infection anxiety associated with working with COVID-19-positive patients and colleagues and fear of exposing themselves, their families and vulnerable patients to infection:

‘Mental exhaustion, profuse worry about myself and my family.’ (Wave I, Medical Doctor, P93)

‘Stressful and uncertain. Always dangerous to come to work.’ (Wave I, Anaesthetic Medical Officer, P7)

‘Keeping the consulting room sanitised and safe makes me anxious in case I am not doing it right.’ (Wave I, Counselling Psychologist, P44)

Particularly during wave I, participants mentioned stress related to resource constraints and a lack of personal protective equipment (PPE) as well as poor adherence to safety procedures by some colleagues and patients:

‘Not having the right PPE when in casualty and being told by superiors that choosing to resuscitate these patients without PPE is my own fault.’ (Wave I, Medical Officer, P106)

‘Patients and staff don’t follow protocol creating dangerous situations.’ (Wave I, General Practitioner, P86)

Although the authors did not specifically ask where participants worked, when discussing stress-related issues, the authors noted differences across HCWs in the private sector compared with those in the public sector. Private HCWs’ stresses often related to loss of income, school closures and hospital access restrictions that meant they could not consult with patients – in some cases, practitioners had lost entire caseloads during the harsh lockdown in wave I:

‘Limited procedures being allowed and the massive reduction in patients places a large financial burden on me, above all the clinical concerns.’ (Wave I, Dentist, P63)

Public HCW’s stress related to poor working conditions, a lack of leadership in some instances, a significant increase in workload and a lack of PPE:

‘Chaos and unorganised. No leadership from management and government doesn’t care about our working environment or safety.’ (Wave I, Physiotherapist, P224)

‘Extremely short-staffed in general medicine at [*tertiary hospital*]. My breaks […][*are*] almost non-existent as keep being pulled out to [*work in*] COVID wards because of [*patient*] numbers and staff shortages.’ (Wave III, Medical Registrar, P607)

As a result of the stress experienced while working during COVID-19, participants across all three waves reported decreased mental and physical health, including symptoms such as headaches, sinus issues, fatigue, poor sleep, muscle pain, back pain, unhealthy eating patterns, emotional and mental exhaustion, burnout, decreased motivation and increased anxiety:

‘I’m always stressed and have outbursts at work.’ (Wave I, Nurse, P117)

‘I have been very tired and had a lot of headaches. I have also found it harder to concentrate and I am finding a lot of office noises (printer and copier working) distracting and annoying.’ (Wave I, Audiologist, P13)

‘Sleep-deprived due to stress and uncertainty, resulting in a weakened immune system.’ (Wave I, Occupational Therapist, P169)

‘The lack of work gives me a feeling of hopelessness and not being purpose-driven. It’s been a bit of a dark space and difficult to get out of.’ (Wave III, Speech Therapist, P548)

In some cases, these symptoms affected work performance significantly. Several participants indicated they had tested positive for COVID-19 themselves, which had also impacted their physical and mental state:

‘Not good, I tested positive for corona and have felt emotionally, mentally and physically drained.’ (Wave I, Occupational Therapist, P150)

‘I was infected with COVID-19 and ended up in ICU.’ (Wave I, Dentist, P55)

These findings on the theme of stress experienced by HCWs during the COVID-19 pandemic confirm those summarised in the literature both locally (Dawood et al. 2022; Naidoo et al. 2020) and internationally (De Kock et al. 2021; Greenberg 2020; Saragih et al. 2021; Spoorthy et al. 2020). Importantly, they offer individual perspectives on the experience of stress across a range of HCWs, in contrast to some of the published literature that tends to focus on frontline workers or on specific professions only.

### Adjustment

A second major theme across the data set related to *adjustment* to the uncertainty that comes with new protocols, workload changes and new ways of working.

The authors noted differences particularly across public and private practitioners regarding experiences of workload and changes to workload during the pandemic. For participants in the public sector, their workload increased substantially, linked in part to staff shortages because of infection and quarantine as well as system failures and resource constraints. In many instances, public HCWs across all three waves were overwhelmed with cases:

‘There is a tense atmosphere due to the increased workload … we [are] just robots.’ (Wave I, Radiographer, P66)

‘An overwhelming experience where there is never enough time or resources to do the work adequately or safely.’ (Wave II, Doctor, P467)

‘Please come and see what is happening in the hospitals right now. We are beyond capacity. We need doctors and nurses and equipment. We need more facilities.’ (Wave III, Doctor, P613)

In other instances, particularly for participants working in private practice and in some medical specialities where routine care and procedures were cancelled during the pandemic, participants experienced a significant decrease in workload and, as mentioned under the previous theme, associated stress because of loss of income:

‘Uncertain of the future of my practice (costs/families with financial strain).’ (Wave II, Speech Therapist, P427)

Participants also reported having to adjust to new protocols and new ways of working. Wearing PPE proved challenging both physically and mentally, leading to increased stress:

‘It’s become stressful and difficult to carry out [*my*] job in PPE, very tiring and difficult to breathe with [*an*] N95 mask.’ (Wave I, Physiotherapist, P202)

‘It has been difficult. One has to constantly adjust and absorb new information.’ (Wave II, Psychiatrist, P414)

In addition to staff shortages and resource constraints, the need to adjust at work has not always been a smooth process for HCWs because of a lack of management or leadership and decision-making in uncertain circumstances:

‘Politics and lack of staff and lack of deep cleaning services. Most of the multidisciplinary team is divided on how to approach some situations, we are often forced to do things we don’t feel comfortable with [*group work*] and when voicing concerns, members of the team are labelled as resistant or lazy which further worsens the level of morale at work.’ (Wave I, Clinical Psychologist, P39)

‘I feel SO bad that patients are being turned away due to a lack of beds, etc. It’s scary to think of how fast this can progress. Many patients’ appointments have had to be cancelled initially and now many are scared to come even though they need it. It’s always a question of “should we be using full PPE or is this patient okay and should I rather save the PPE for another patient”.’ (Wave I, Speech Therapist and Audiologist, P402)

Several participants in wave I, particularly allied HCWs, mentioned challenges related to adjusting to the swift transition to telehealth during this time. Some participants worried about the quality of care they were able to provide online. In particular, they mentioned a loss of connection with patients and a sense that online treatment was not the same as face-to-face treatment:

‘Online therapy [*is*] strangely intense and exhausting … I have felt deeply sad at the loss of face-to-face contact with patients and the old rhythms of greeting and meeting in the waiting room and farewell at the door.’ (Wave I, Counselling Psychologist, P44)

‘There is a lot more planning and prep needed for [*online*] sessions. This takes more of my time, then resulting [*in*] having less time for other things.’ (Wave III, Speech Therapist, P542)

Many participants spoke about the uncertainty that the pandemic had brought to all aspects of their lives. Among wave II and particularly wave III participants, the authors observed a shift in perspective from the initial uncertainty and heightened stress brought on by wave I, towards a sense of resignation that uncertainty during the pandemic is inevitable. However, some participants indicated that by the time waves II and III arrived, they felt better prepared mentally to cope with working during the pandemic. Participants oriented to the need for flexibility and adaptability during the pandemic – and saw these aspects as crucial to coping with the uncertainty of work:

‘Need to be flexible and adaptable. Changes can permit creativity and thinking outside the box.’ (Wave II, Speech Therapist, P427)

‘I feel like I have been able to wrap my head around most lockdown [*and*/*or*]COVID stuff over the past few months. I feel more prepared mentally for wave 2 … perhaps not better prepared emotionally. I have realised that everyone is going through the same thing (same storm, but perhaps [*a*] different boat), and that I need to be patient with others.’ (Wave II, Speech Therapist, P429)

These findings on the theme of adjustment confirm what other local authors have reported that HCWs found it difficult to cope with the large numbers of patients presenting with COVID-19 at South African hospitals (Burger & Mchenga 2021). They also confirm the findings of international studies (Grailey et al. 2021), specifically that HCWs experienced challenges with adjusting to new barriers in their work such as the high workload associated with the pandemic and the impact of PPE. On the topic of HCWs’ challenges related to the transition to online therapy, the findings align with some international studies that have highlighted challenges with developing an HCW–patient relationship online because of the loss of physical connection (Gomez et al. 2021).

### Support

A third major theme across the data set was that of *support*, in terms of support experiences and support needs during the pandemic. Although HCWs experienced stress during the pandemic, support from colleagues seemed central to alleviating anxieties and mitigating uncertainty.

Many participants indicated that they received support at work during the COVID-19 pandemic, largely from colleagues and management or via debrief sessions, employee wellness programmes, peer supervision as well as professional organisations:

‘My team is extremely close and we talk to each other about everything. Our hospital has daily debriefing sessions for those who need it.’ (Wave I, Occupational Therapist, P80)

‘Fellow colleagues are sometimes willing to share burdens; the juniors have been the most open to supporting and needing support.’ (Wave II, Anaesthetist, P524)

Other respondents felt that they did not receive any support at work, particularly because of poor management practices. Importantly, these sentiments remained a strong feature of the wave III data set:

‘I don’t feel respected or cared for. My employer rejected a shift option and now if one of our multidisciplinary team members … is affected by the virus, we will all be affected at once and our whole department would have to shut down leaving our patients without rehab services.’ (Wave I, Audiologist, P87)

‘I’ve reached out to seniors when I couldn’t cope and I was met with a very hostile, unsympathetic response that has left me more angry than anything. Everyone claims to be of support but when you actually need it. There’s nothing.’ (Wave III, Community Service Doctor, P597)

Those in private practice felt they lacked support largely because they work on their own, although in some instances, colleagues in the field and professional organisations became sources of support:

‘Very little. As a business owner working alone the responsibility is yours entirely.’ (Wave I, Dentist, P63)

Importantly, the support offered at work often seemed ad hoc rather than formalised. Colleagues initiated support structures among each other in the absence of managerial support in some cases and work WhatsApp groups played a key role in this regard.

Support outside of the workplace generally came from family, friends, pets, hobbies and in a few cases, even from domestic helpers in the home environment. Religious and social networks were also mentioned as helpful in this regard. Participants indicated that having a family member or partner in the same line of work was a source of great support since that person understood their work situation:

‘My husband is also a health care worker and understands the situation.’ (Wave I, Physiotherapist, P274)

‘At home, the support through family prayer is more than enough for me.’ (Wave II, Health and Safety Officer, P455)

Importantly, a few participants mentioned not having access to any support outside the work environment. In several instances, mental health challenges among other family members at home meant that instead of receiving support, HCWs were the ones giving the support:

‘… it is sometimes difficult to speak to people who don’t understand the context and so talking at home is sometimes not as helpful.’ (Wave I, Occupational Therapist, P167)

‘None, my son attempted suicide and my husband and daughter lost their jobs.’ (Wave I, Physiotherapy Manager, P358)

‘Spouse who ignores what is happening. Won’t talk about it. Teenagers with their issues. Depression Anxiety. Lash out at me. Full time work plus household chores, kids. Homework. Exhausted.’ (Wave II, Doctor, P485)

‘I stay with my wife. We mostly don’t see my and her parents for fear of passing on the virus to them. Last year I didn’t see my parents for around 10 months. So, my wife is my support mechanism at home. She has been struggling with depression herself, so it’s hard to be too reliant on her.’ (Wave III, Doctor, P613)

In the wave III data, in particular, there was a tendency among participants to talk about the need to take charge of their own mental health care and find ways to engage in self-care:

‘Take it one day at a time. Self-care and self-love is key to a healthy mental status. Keep calm and avoid panic.’ (Wave III, Dietician, P610)

‘I’ve learnt from this year and this pandemic that you’re truly on your own and must look after yourself.’ (Wave III, Community Service Doctor, P597)

The authors also observed an acknowledgement that professional help is required in addition to finding one’s own avenues for self-care. Participants spoke about how an overreliance on family for support can contribute to their burnout, hence the need to seek support elsewhere. Some participants in wave III also demonstrated a shift from the need to receive support towards becoming a support structure for others in the work environment:

‘No real emotion support given at work, I have seeked [*sic*] out support from a counsellor.’ (Wave III, Speech Therapist, P592)

‘I am the support others look to.’ (Wave III, Speech Therapist, P549)

Many participants specifically mentioned the need for psychological support for all HCWs, not just for those traditionally considered as ‘frontline’ workers (doctors and nurses). In general, they felt the Department of Health has a responsibility to support both public and private sector HCWs, be it psychologically or in terms of providing more resources:

‘Don’t forget about healthcare workers who are not on the front lines. Just because we are not treating COVID patients directly doesn’t mean that we aren’t struggling too. Assisting our patients is very different now, and the patients are needing a lot more support from us, which takes a lot of energy and can be very draining.’ (Wave I, Audiologist, P22)

‘You need to support health care workers as they are terrified and overworked.’ (Wave I, Clinical Pharmacist, P33)

The authors noted many responses across all three waves related to the government’s failure to adequately provide resources during the pandemic. In the wave III dataset in particular, there was a strong sense of frustration and anger among many participants in their assertions that the government has failed HCWs during the pandemic – through a lack of transparency and trust, resource constraints and the slow vaccine rollout:

‘Government needs to account for its poor response and provide honest and transparent leadership. The vaccine debacle is appalling. They blatantly lied because they have no plan for vaccination. I lived through HIV patients being denied ART and now a similar unforgivable response to COVID vaccination.’ (Wave II, Specialist Physician, P502)

‘… why has the government stolen SO MUCH MONEY during COVID. Those funds were meant for us as HCWs and the rest of the country … Maybe stop stealing, come to the ground and see what your citizens are ACTUALLY going through. Do more and do better. We deserve this for putting our lives on the line DAILY.’ (Wave III, Speech Therapist and Audiologist, P584)

Several participants across all three waves indicated a feeling of abandonment coupled with a sense of not feeling heard, valued or understood by the general public and the Department of Health:

‘Mostly feel abandoned by management and [*provincial*] Department of Health who don’t seem to care about the lack of resources.’ (Wave III, Doctor, P613)

‘Come and spend a night in a busy COVID casualty or emergency unit and see for yourself. You won’t understand what needs to be improved unless you’re here … When I’m finished community service, I’m leaving public health for good. I can’t handle it and I so badly wanted to work here.’ (Wave III, Doctor, P597)

‘Communicate clearly and make people feel heard and safe.’ (Wave II, Psychiatrist, P410)

‘Value your health care workers going forward and after this is finally over, remember the role they played and how despite zero resources at the worst of times, they still showed up. With no strikes or protests and little emotional support.’ (Wave II, Physiotherapist, P516)

These findings on support mechanisms and strategies confirm those of international studies such as that of Grailey et al. (2021), which highlight differences in the types of psychological support received and accessed by HCWs during the pandemic. They also confirm what local authors such as Curran et al. (2021) highlight, that there is an urgent need for psychological support for HCWs during and after this pandemic.

## Discussion

A universal thread of uncertainty seems central to HCWs’ experiences during COVID-19. Work during the pandemic has been a stressful time for HCWs in both private and public healthcare sectors in South Africa – as borne out in the quantitative results of the broader survey described elsewhere. The results of our study confirm those of international and local literature, highlighting increased reported levels of stress and anxiety among HCWs during the pandemic and confirming calls for psychological support for HCWs.

Underlying many of the responses from participants across all three waves are references to pressures in the South African healthcare system, particularly the public system, which have been exacerbated by the COVID-19 pandemic. Limited resources, together with the overwhelming load that the pandemic has placed on an already fragile healthcare system, appear to have played a key role in exacerbating the stress experienced by HCWs during this time. Although there were distinctions in experiences noted across public versus private healthcare sectors, there were commonalities in terms of stress, uncertainty, the need for adjustment and the need for support.

The three major qualitative themes identified in the data intersect and influence each other: for example, adjustment to workload issues can lead to stress; the availability (or lack thereof) of support systems influences how HCWs respond to stress and adjust to working during the pandemic. Although the authors identified several common themes across the data sets, the findings also point to diversity across experiences – for example, some HCWs have had access to strong support structures while others have not – suggesting the need for tailored responses.

Several implications emerge from this study, not just for the South African context but for international contexts too. Firstly, there is a clear need for workplaces and professional organisations to proactively offer wellbeing and mental health support to *all* HCWs (and indeed all people working in healthcare spaces), not just to those traditionally defined as frontline workers. Importantly, these structures need to be contextually relevant and may be more acceptable if driven in a bottom-up way by HCWs themselves rather than via a top-down managerial approach. Although informal, organic approaches that have developed among HCW groups (including WhatsApp support and debrief groups) are useful in this regard, there are limitations to the amount and depth of support they can offer in response to the complex experiences and support needs of HCWs. As our findings show particularly in the wave III data, there is a shift among HCWs themselves towards acknowledging the need for professional psychological assistance. This shift is an important one that needs to be nurtured and encouraged in light of the reluctance of many HCWs to seek professional support during the pandemic (Galbraith et al. 2021) and the assumption that HCWs should be able to ‘fix themselves’ (The Mercury 2022).

Our findings give voice to the experiences of HCWs and confirm the basic need for them to feel heard and valued (Dawood et al. 2022) – not only in their workplaces but also at home by their families and more broadly by the government and the general public. Here lies an opportunity for a range of creative solutions to be pursued to address this need. For example, online platforms could be created for HCWs to share their stories and experiences and thus put a public ‘face’ to the many HCWs who have worked tirelessly during this pandemic. Such solutions are easily implementable by a range of stakeholders. Importantly, HCWs are going to require continued support beyond the COVID-19 pandemic given the significant stresses they have experienced.

## Conclusion

This study has several limitations. The number of participants in each data set differed considerably across the waves – with a decrease in participation across the waves likely because of general trends of survey fatigue noted during the pandemic (De Koning et al. 2021). The high number of female participants may indicate that they were more affected by the pandemic and thus more inclined to complete the survey. The sample was not evenly distributed across types of occupations, with the sample skewed towards allied HCWs. Reasons for this are not clear and may relate to which of the professional bodies actually distributed our survey invitation to their members. Also, the authors neglected to collect data on the province in which participants work.

Regardless of these limitations, this study offers a rich, unique perspective into the experiences and voices of a wide range of HCWs across several waves of the COVID-19 pandemic in South Africa. It presents some key findings and implications that are important not only in this context but around the world too, relevant both now and for future pandemic preparedness. Subsequent research involving in-depth follow-up interviews with a small sample of HCWs from this study (Kazadi 2022) confirms that the experiences of HCWs working during COVID-19 are diverse and complex and that there is an urgent need to find ways to address the ongoing impact of the COVID-19 pandemic on HCWs.
