# Corrigendum: The mental health and well-being of healthcare workers during COVID-19 in South Africa

**DOI:** 10.4102/hsag.v29i0.2621

**Published:** 2024-05-06

**Authors:** Jennifer Watermeyer, Sonto Madonsela, Johanna Beukes

**Affiliations:** 1 Health Communication Research Unit, School of Human and Community Development, Faculty of Humanities, University of the Witwatersrand, Johannesburg, South Africa

In the published article, Watermeyer, J., Madonsela, S. & Beukes, J., 2023, ‘The mental health and wellbeing of healthcare workers during COVID-19 in South Africa’, *Health SA Gesondheid* 28(0), a2159. https://doi.org/10.4102/hsag.v28i0.2159, the authors neglected to include the funder.

Instead of:

## Funding information

This research received no specific grant from any funding agency in the public, commercial or not-for-profit sectors.

It should be:

## Funding information

This work was supported by the National Institute for the Humanities and Social Sciences (NIHSS), (no specific grant number attached to this funding) to J.B.

The authors apologise for this error. The correction does not change the significance of the study’s findings or overall interpretation of the study’s results or the scientific conclusions of the article in any way.

